# Supplementary material for: Transcriptional diversification in a human-adapting zoonotic pathogen drives niche-specific evolution
Source: Nat Commun. 2025 Feb 28;16:2067. doi: 10.1038/s41467-025-57331-6 (PMC11871327; doi:10.1038/s41467-025-57331-6)
Supplement: Supplementary file 1 — Supplementary Information [file 41467_2025_57331_MOESM1_ESM.pdf]

SUPPLEMENTARY INFORMATION

Transcriptional Diversification in a Human-Adapting Zoonotic Pathogen Drives Niche-Specific Evolution

Soma Ghosh[1*], Chao-Jung Wu[1,a,*], Abraham G. Moller[1], Adrien Launay[1,b], Laina N. Hall[2,c], Bryan T. Hansen[2], Elizabeth R. Fischer[2], Jung-Ho Youn[3], Pavel P. Khil[1,3], John P. Dekker[1,3,#]

[1]Bacterial Pathogenesis and Antimicrobial Resistance Unit, Laboratory of Clinical Immunology and Microbiology, National Institute of Allergy and Infectious Diseases, National Institutes of Health, Bethesda, MD; [2]Research Technologies Branch, Rocky Mountain Laboratories, National Institute of Allergy and Infectious Diseases, National Institutes of Health, Hamilton, MT; [3]National Institutes of Health Clinical Center, National Institutes of Health, Bethesda, MD.

*Equal contribution

[a] Present address: School of Medical Laboratory Science and Biotechnology, College of Medical Science and Technology, Taipei Medical University, Taipei 110301, Taiwan; [b]Present address: Endogenomiks, Zapopan, Jalisco, Mexico; [c] Present address: University of California Berkeley, Berkeley, CA

[#]Address correspondence to John Dekker: john.dekker@nih.gov

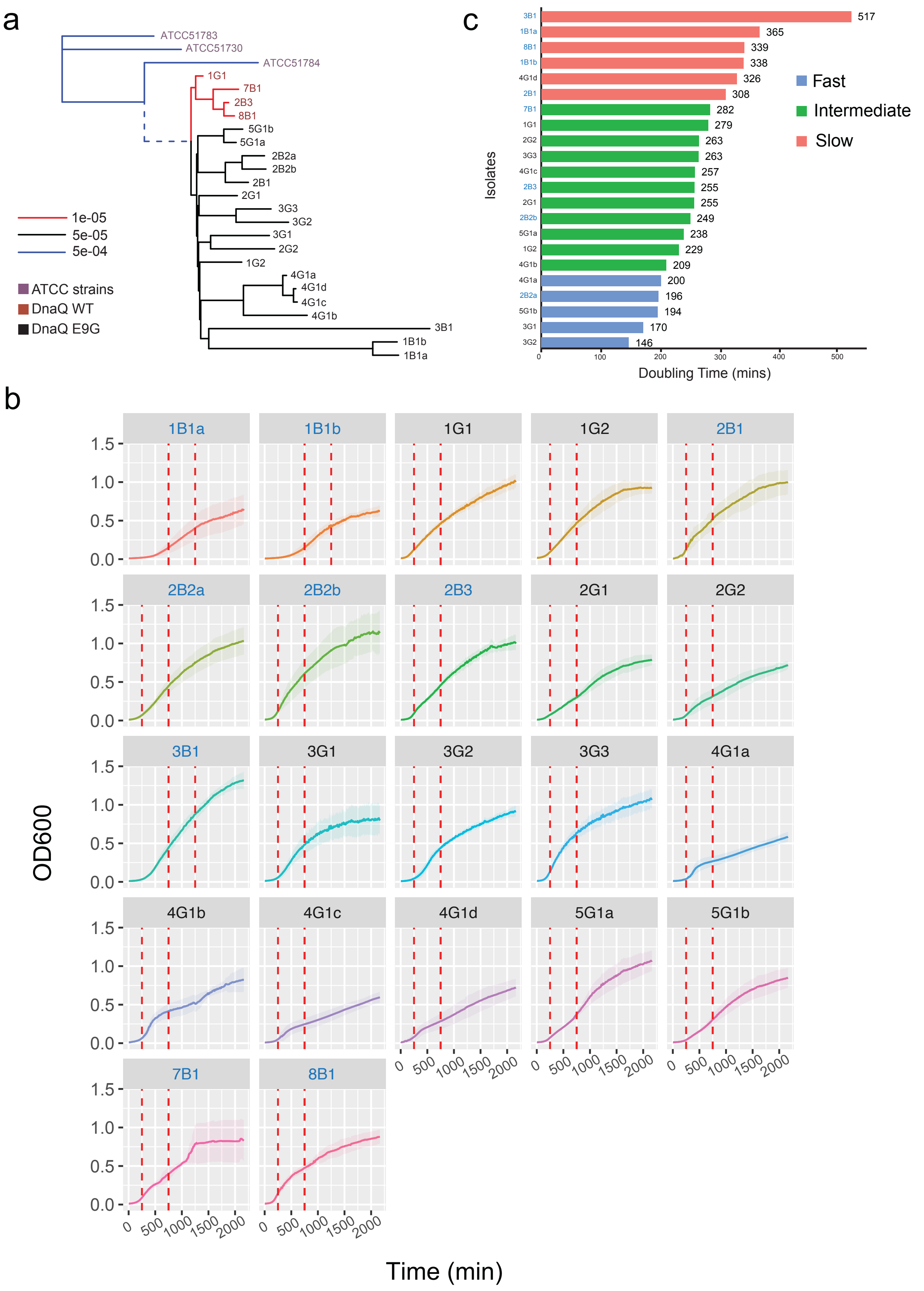
a
ATCC51783
ATCC51730
ATCC51784
1G1
7B1
2B3
8B1
5G1b
5G1a
2B2a
2B2b
2B1
2G1
3G3
3G2
3G1
2G2
1G2
4G1a
4G1d
4G1c
4G1b
3B1
1B1b
1B1a
1e-05
5e-05
5e-04
ATCC strains
DnaQ WT
DnaQ E9G
c
Isolates
3B1 517
1B1a 365
8B1 339
1B1b 338
4G1d 326
2B1 308
7B1 282
1G1 279
2G2 263
3G3 263
4G1c 257
2B3 255
2G1 255
2B2b 249
5G1a 238
1G2 229
4G1b 209
4G1a 200
2B2a 196
5G1b 194
3G1 170
3G2 146
0
100
200
300
400
500
Doubling Time (mins)
Fast
Intermediate
Slow
b
OD600
1B1a
1B1b
1G1
1G2
2B1
2B2a
2B2b
2B3
2G1
2G2
3B1
3G1
3G2
3G3
4G1a
4G1b
4G1c
4G1d
5G1a
5G1b
7B1
8B1
0.0
0.5
1.0
1.5
0
500
1000
1500
2000
Time (min)

Fig S1. Phylogenetic relationship between the isolates used in this study. a) Maximum likelihood phylogenetic tree of 22 clinical *B. hinzii* isolates and three ATCC strains constructed based on the core genome sequences of the isolates (see Methods for details). Clinical isolates with WT DnaQ and E9G DnaQ are indicated by red and black branches, respectively. The ATCC strains are indicated by blue branches. b) Growth curves of all isolates in LB medium. Data represent a total of 3 biological replicates, each with 4 technical replicates (n=12 measurements). The solid line represents mean values across all biological and technical replicates and standard deviation is represented with shading. The vertical lines indicate the interval selected for doubling time calculations. Blood isolates are indicated in blue and GI isolates are shown in black. c) Calculated doubling times of all isolates grown in LB medium. Blue bars indicate isolates with doubling times of 200 minutes or less (“fast” growth), green indicate doubling times between 200 and 300 minutes (“intermediate” growth), and pink indicates doubling time greater than 300 minutes (“slow” growth).

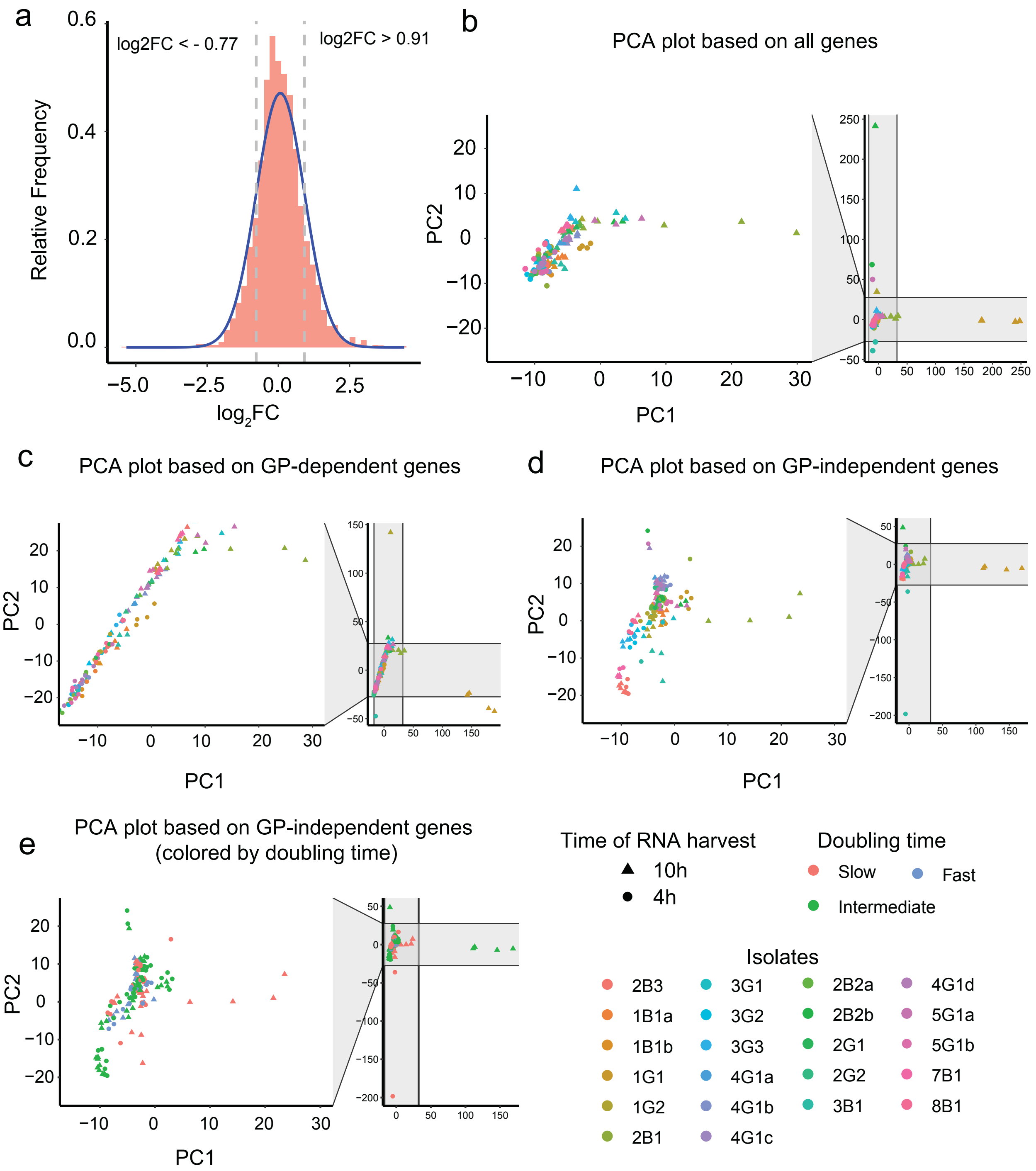

Fig S2. Identification of growth phase dependent genes. a) Density plot of log_2FC calculated between 4hr and 10hr timepoints in isolate 2B3 is shown. The distribution was fitted to a gaussian curve (blue) to identify cutoff at Z-score = ±1 (dashed lines). Genes outside the cutoff were classified as growth phase (GP) dependent genes. b) PCA plot of the 153 transcriptomes based on all genes, colored by isolate (color scale

at bottom right under “Isolates”). Circles indicate the 4hr time points and triangles indicate 10hr time points. c) PCA plot based on GP dependent genes, colored by isolate (color scale at bottom right under “Isolates”). Circles indicate the 4hr time points and triangles indicate 10hr time points. d) PCA plot for GP independent genes, colored by isolate (color scale at bottom right under “Isolates”). Circles indicate the 4hr time points and triangles indicate 10hr time points. e) PCA plot based on GP independent genes, colored based on their doubling time (color scale at bottom right under “Doubling time”). Circles indicate the 4hr time points and triangles indicate 10hr time points.

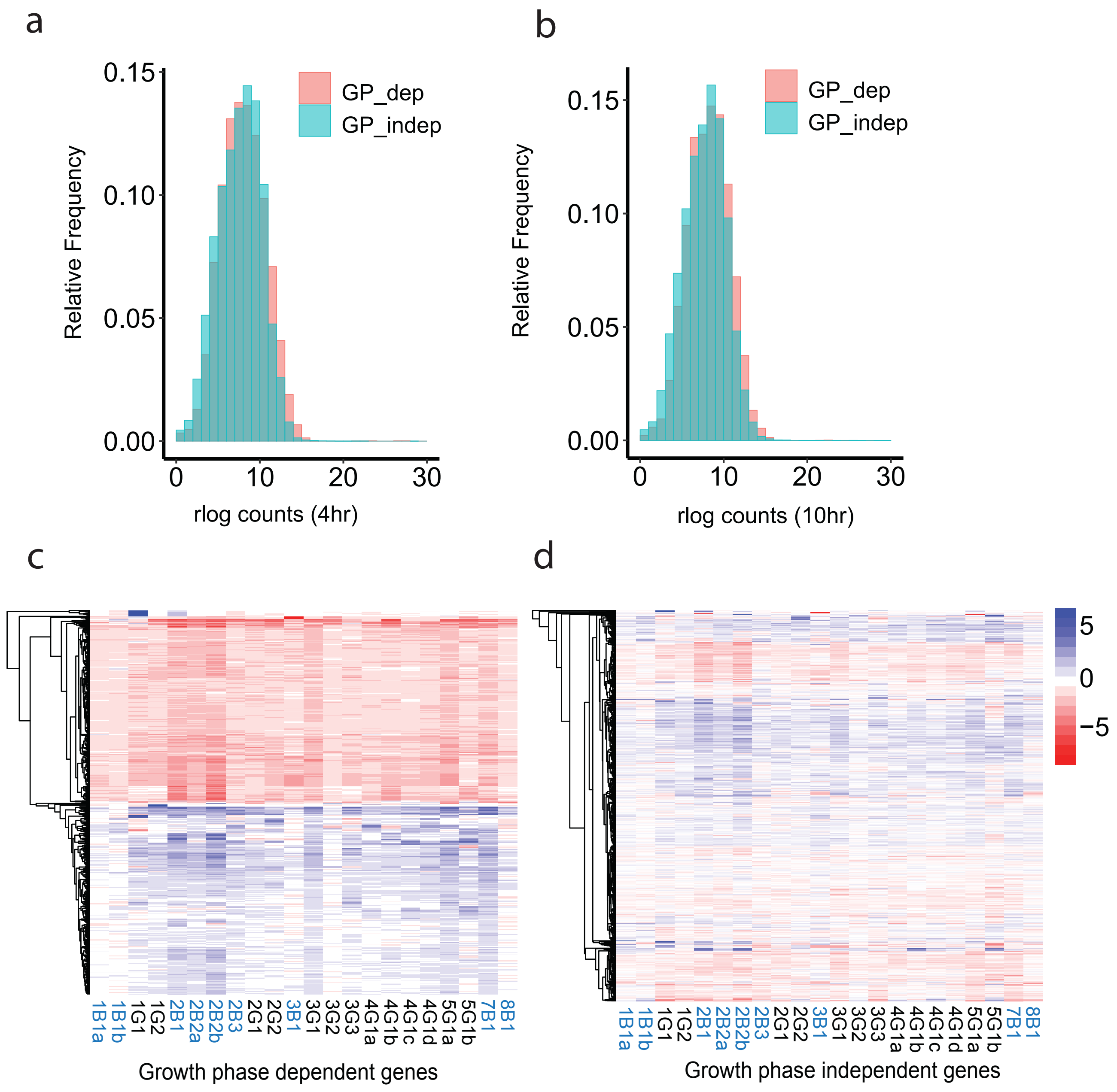

Fig S3. Comparison of growth phase dependent and independent gene sets. Relative distribution of rlog count values for GP dependent and GP independent genes at a) 4hr and b) 10hr time points. Differences in the normalized logarithmic counts between 4hr and 10hr timepoints were calculated and plotted as heatmap for c) GP dependent and d) GP independent gene sets for all clinical isolates. Color represents count differences normalized between -8 to 8.

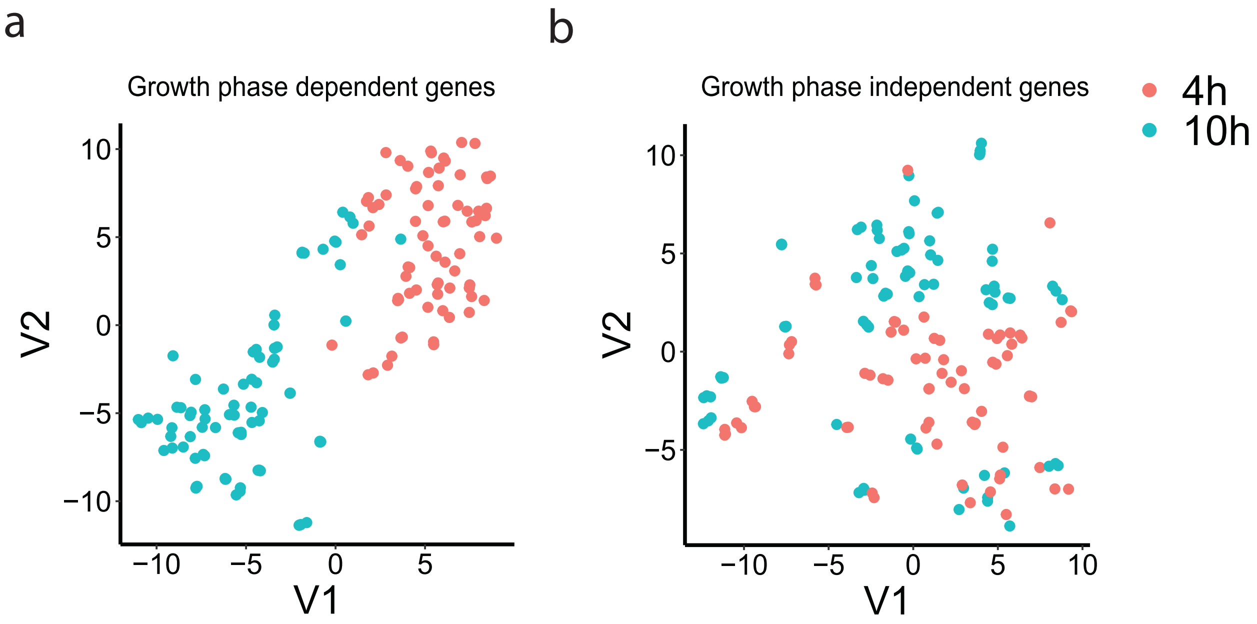

Fig S4. Growth phase dependent genes display clear temporal separation, which is reduced in growth phase independent gene set. tSNE plot of 153 transcriptomes were generated based on a) GP dependent and b) GP independent gene sets. The samples are colored based on the time point to indicate time dependent separation. Plots were prepared in R using the Rtsne package.

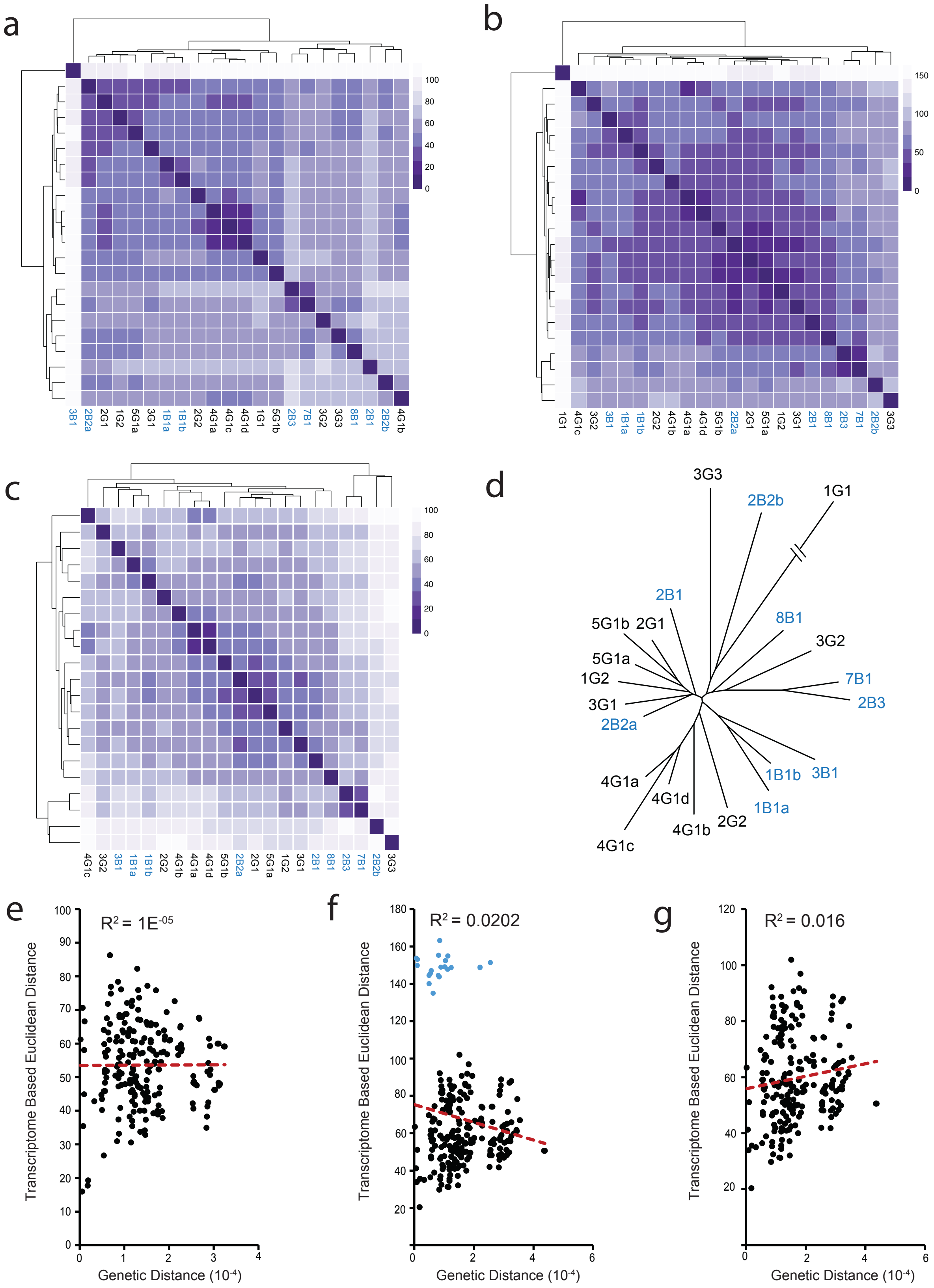
a
3B1
2B2a
2G1
1G2
5G1a
3G1
1B1a
1B1b
2G2
4G1a
4G1c
4G1d
1G1
5G1b
2B3
7B1
3G2
3G3
8B1
2B1
2B2b
4G1b
100
80
60
40
20
0
b
1G1
4G1c
3G2
3B1
1B1a
1B1b
2G2
4G1b
4G1a
4G1d
5G1b
2B2a
2G1
5G1a
1G2
3G1
2B1
8B1
2B3
7B1
2B2b
3G3
150
100
50
0
c
4G1c
3G2
3B1
1B1a
1B1b
2G2
4G1b
4G1a
4G1d
5G1b
2B2a
2G1
5G1a
1G2
3G1
2B1
8B1
2B3
7B1
2B2b
3G3
100
80
60
40
20
0
d
3G3
2B2b
1G1
2B1
5G1b
2G1
8B1
3G2
5G1a
1G2
7B1
3G1
2B3
2B2a
1B1b
3B1
4G1a
4G1d
1B1a
2G2
4G1b
4G1c
e
$R^2 = 1E^{-05}$
Transcriptome Based Euclidean Distance
Genetic Distance (10^{-4})
f
$R^2 = 0.0202$
Transcriptome Based Euclidean Distance
Genetic Distance (10^{-4})
g
$R^2 = 0.016$
Transcriptome Based Euclidean Distance
Genetic Distance (10^{-4})

Fig S5. Isolates display exceptional transcriptional diversity. Clustered heat map of Euclidean distances between the transcriptomes (only GP independent genes) of all isolate pairs at a) 4 hr time point, b) 10 hr time point, and c) 10 hr time point after removing the outlier 1G1. The color of each cell represents the normalized Euclidean distance between the two compared isolates, as indicated by the bar at right. d) Euclidean distances for 10 hr time point (from part b) were used to generate a neighbor joining tree to represent relationships in transcriptome space. e) Correlation plot with a linear fit between the genotypic distance as determined using RaXML and the transcriptomic distance between pairs of isolates at 4hr time point after removing the outlier 3B1. f) Correlation plot with a linear fit between the genotypic distance as determined using RaXML and the transcriptomic distance between pairs of isolates at 10hr time point. 1G1 (outlier) are shown in blue dots. g) Correlation plot with a linear fit between the genotypic distance as determined using RaXML and the transcriptomic distance between pairs of isolates at 10hr time point after removing the outlier 1G1.

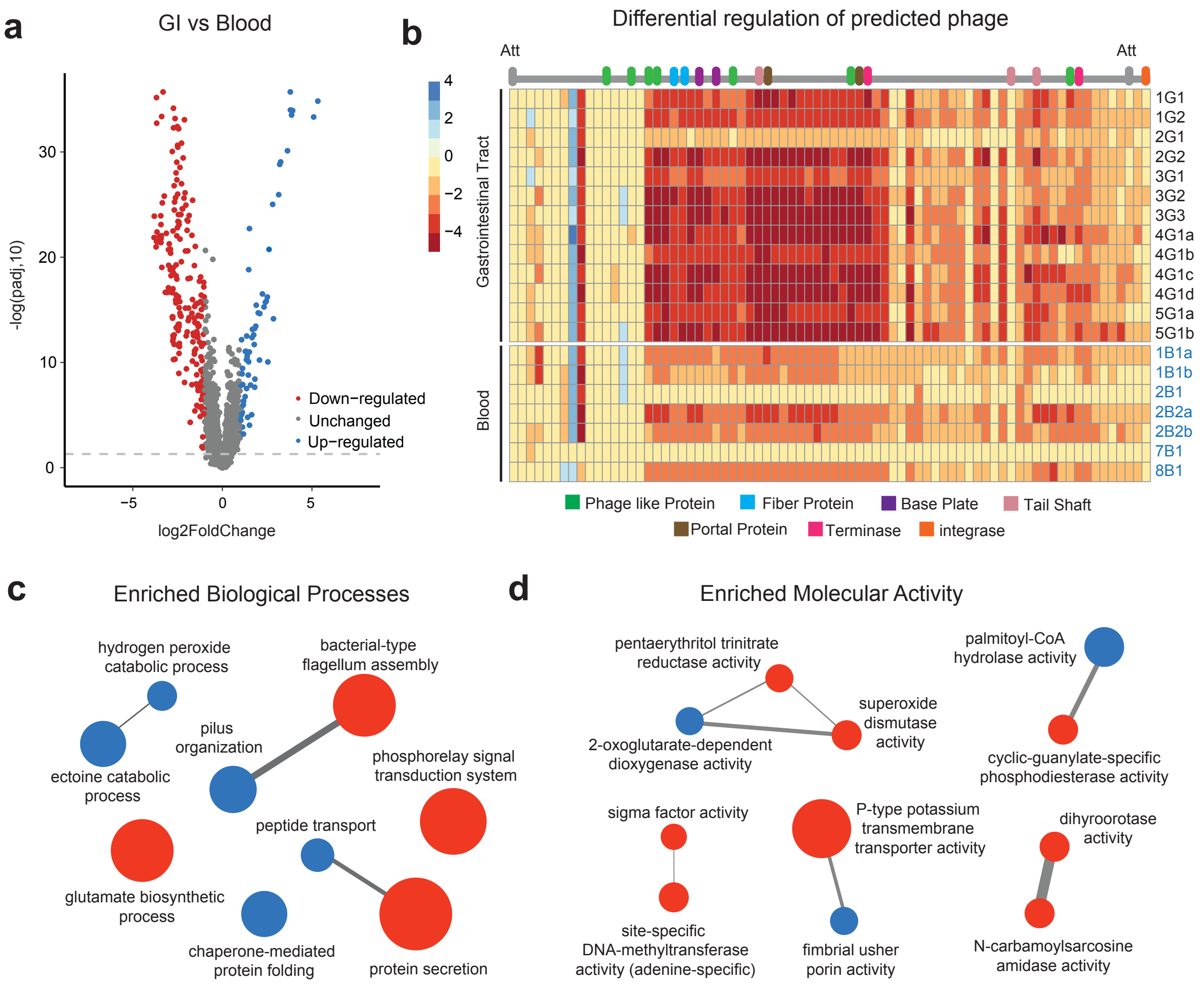

Fig S6. Niche specific differential gene expression in isolates derived from blood and gastrointestinal tract. a) Volcano plot displaying differentially expressed genes between groups of isolates harvested from blood and the GI tract. Red represents genes that were downregulated in GI isolates when compared to isolates derived from the blood. b) Heatmap representing the fold change in genes annotated as a putative phage, phiEt88 (columns) for each isolate (rows). Phage specific genes as annotated by PHASTER (see Methods) are color coded and shown in the top panel. Heat map values range from 4 to -4 and represent log_2FC with reference to 2B3. Gene Ontology analysis of differentially expressed genes identified c) enriched biological processes and d) molecular activity between the two groups. Red represents genes/ontologies that are downregulated in GI isolates, while blue represents gene and ontologies that are upregulated in GI isolates. Node size represents statistical significance for the specific GO term with larger nodes representing lower p-values. Only nodes with adjusted p-value < 0.05 are represented on the network. Relation between Gene Ontology terms was derived from REVIGO.

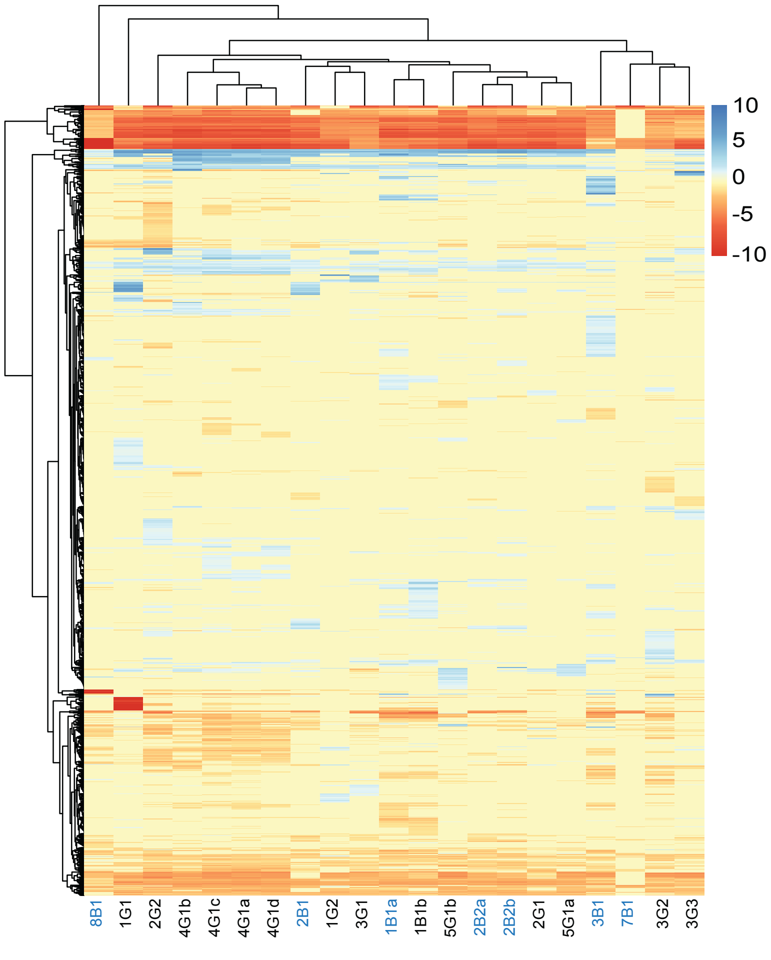

Fig S7. Clustered heat map of log_2FC values of all genes in all isolates as compared to reference isolate, 2B3.
