# Supplementary material for: Transcriptional diversification in a human-adapting zoonotic pathogen drives niche-specific evolution
Source: Nat Commun. 2025 Feb 28;16:2067. doi: 10.1038/s41467-025-57331-6 (PMC11871327; doi:10.1038/s41467-025-57331-6)
Supplement: Supplementary file 2 — Description of Additional Supplementary Files [file 41467_2025_57331_MOESM2_ESM.pdf]

Description of Additional Supplementary Files

Supplemental Data 1. Details of the sequencing libraries used in the study and culture OD600 values for all RNA harvests.

Supplemental Data 2. a) log2FC values of genes between 4hr and 10hr time points in 2B3. b) List of genes identified as growth phase independent, and c) as growth phase dependent based on log2FC values.

Supplemental Data 3. a) rlog counts for all genes in all isolates as determined from DEseq2 analysis at 4hr time point. b) rlog counts for all genes in all isolates as determined from DEseq2 analysis at 10hr time point.

Supplemental Data 4. a) rlog count differences between the 10hr time point and 4hr time point for growth phase dependent genes. b) rlog count differences between the 10hr time point and 4hr time point for growth phase independent genes.

Supplemental Data 5. a) Euclidean distances between pairs of isolates using rlog count values for growth phase independent genes at 4hr time point. b) Euclidean distances between pairs of isolates using rlog count values for growth phase independent genes at 10hr time point.

Supplemental Data 6. Differential expression (log2FC values) of GP independent genes between isolates cultured from blood and GI tract. Differentially expressed genes are defined as those having log2FC > 1 or <-1 and padj <0.05. Log2FC = 0 refers to cases that were not statistically significant and thus the genes were not considered differentially expressed.

Supplemental Data 7. log2FC values of growth phase independent in each isolate with reference to 2B3. Differentially expressed genes are defined as those having log2FC > 1 or <-1 and padj <0.05. Log2FC = 0 refers to cases that were not statistically significant and thus the genes were not considered differentially expressed.

Supplemental Data 8. Summary Nanodisco methylation motif analysis for all isolates.

Supplemental Data 9. Differentially expressed genes shared by 1B1a and 1B1b with reference to 2B3.

Supplemental Data 10. Gene ontology terms that are enriched in individual isolates relative to reference isolate 2B3.
