# Supplementary material for: Transcriptional diversification in a human-adapting zoonotic pathogen drives niche-specific evolution
Source: Nat Commun. 2025 Feb 28;16:2067. doi: 10.1038/s41467-025-57331-6 (PMC11871327; doi:10.1038/s41467-025-57331-6)
Supplement: Supplementary file 12 — Reporting Summary [file 41467_2025_57331_MOESM12_ESM.pdf]

nature portfolio

Corresponding author(s): John P. Dekker

Last updated by author(s): Jan 18, 2025

Reporting Summary

Nature Portfolio wishes to improve the reproducibility of the work that we publish. This form provides structure for consistency and transparency in reporting. For further information on Nature Portfolio policies, see our Editorial Policies and the Editorial Policy Checklist.

Statistics

For all statistical analyses, confirm that the following items are present in the figure legend, table legend, main text, or Methods section.

n/a	Confirmed	
☐	☒	The exact sample size (*n*) for each experimental group/condition, given as a discrete number and unit of measurement
☐	☒	A statement on whether measurements were taken from distinct samples or whether the same sample was measured repeatedly
☐	☒	The statistical test(s) used AND whether they are one- or two-sided<br>*Only common tests should be described solely by name; describe more complex techniques in the Methods section.*
☒	☐	A description of all covariates tested
☐	☒	A description of any assumptions or corrections, such as tests of normality and adjustment for multiple comparisons
☐	☒	A full description of the statistical parameters including central tendency (e.g. means) or other basic estimates (e.g. regression coefficient) AND variation (e.g. standard deviation) or associated estimates of uncertainty (e.g. confidence intervals)
☐	☒	For null hypothesis testing, the test statistic (e.g. *F*, *t*, *r*) with confidence intervals, effect sizes, degrees of freedom and *P* value noted<br>*Give P values as exact values whenever suitable.*
☒	☐	For Bayesian analysis, information on the choice of priors and Markov chain Monte Carlo settings
☒	☐	For hierarchical and complex designs, identification of the appropriate level for tests and full reporting of outcomes
☒	☐	Estimates of effect sizes (e.g. Cohen's *d*, Pearson's *r*), indicating how they were calculated

Our web collection on statistics for biologists contains articles on many of the points above.

Software and code

Policy information about availability of computer code

Data collection	Standard Illumina instrument software on Illumina Nextseq instrument
Data analysis	MinKNOW 21.05.8, Guppy 5.0.11, TrimGalore 0.6.7, flye 2.9.1, Pilon 1.23,TrimGalore v0.6.6, BWA v0.7.17, Samtools v1.13, Data analysis ctd: HTSeq v0.11.4, InterProScan v5.42-78.0, blast2go basic, topGO v2.40.0, nanodisco1.0.3_dev, compress_fast5 4.0.0, DESeq2

For manuscripts utilizing custom algorithms or software that are central to the research but not yet described in published literature, software must be made available to editors and reviewers. We strongly encourage code deposition in a community repository (e.g. GitHub). See the Nature Portfolio guidelines for submitting code & software for further information.

Data

Policy information about availability of data

All manuscripts must include a data availability statement. This statement should provide the following information, where applicable:

- Accession codes, unique identifiers, or web links for publicly available datasets
- A description of any restrictions on data availability
- For clinical datasets or third party data, please ensure that the statement adheres to our policy

All raw RNA-seq sequencing read files generated in this study have been submitted to SRA with Accession ID PRJNA752389 and GEO ID GSE181542. All base called long read and short read sequences for the ATCC genomes generated in this study have been submitted to ID PRJNA625574. Additional raw data (including

methylation data) generated in this study and R markup files have been uploaded to Zenodo with https://zenodo.org/records/13988484. Supplemental Data File 8 was also uploaded to Zenodo at the DOI above due to size (too large to upload to publication server).

Research involving human participants, their data, or biological material

Policy information about studies with human participants or human data. See also policy information about sex, gender (identity/presentation), and sexual orientation and race, ethnicity and racism.

Reporting on sex and gender	N/A
Reporting on race, ethnicity, or other socially relevant groupings	N/A
Population characteristics	N/A
Recruitment	N/A
Ethics oversight	Informed written consent was obtained from the patient under NIH IRB protocol 93-I-0119 upon admission to the NIH Clinical Center, approved by the NIH IRB committee. Diagnostic clinical cultures were performed as part of routine standard-of-care management under this consented protocol, and only de-identified subcultured bacterial isolates were used in the work presented in this manuscript. The results of the work in this manuscript were not used for patient care. The work presented in this manuscript was thereby excluded from further NIH IRB review, on the basis of the fact that it was a study of a single case, involving only sequencing and analysis of de-identified bacterial isolates.

Note that full information on the approval of the study protocol must also be provided in the manuscript.

Field-specific reporting

Please select the one below that is the best fit for your research. If you are not sure, read the appropriate sections before making your selection.

☒ Life sciences ☐ Behavioural & social sciences ☐ Ecological, evolutionary & environmental sciences

For a reference copy of the document with all sections, see nature.com/documents/nr-reporting-summary-flat.pdf

Life sciences study design

All studies must disclose on these points even when the disclosure is negative.

Sample size	153 RNA-seq transcriptomes, including biological replicates as described in Methods and 22 methylomes
Data exclusions	Failed sequencing libraries not meeting quality control standards were excluded.
Replication	RNA-seq libraries were generated from 25 isolates at one or more time points in biological replicates of 3-5 as described in Methods.
Randomization	N/A
Blinding	No blinding was applied during experiments or analysis.

Reporting for specific materials, systems and methods

We require information from authors about some types of materials, experimental systems and methods used in many studies. Here, indicate whether each material, system or method listed is relevant to your study. If you are not sure if a list item applies to your research, read the appropriate section before selecting a response.

Materials & experimental systems

n/a	Involved in the study	
☒	☐	Antibodies
☒	☐	Eukaryotic cell lines
☒	☐	Palaeontology and archaeology
☒	☐	Animals and other organisms
☒	☐	Clinical data
☒	☐	Dual use research of concern
☒	☐	Plants

Methods

n/a	Involved in the study	
☒	☐	ChIP-seq
☒	☐	Flow cytometry
☒	☐	MRI-based neuroimaging

Plants

Seed stocks	N/A
Novel plant genotypes	N/A
Authentication	N/A
